# Supplementary material for: In Situ LSPR Sensing of Secreted Insulin in Organ-on-Chip
Source: Biosensors (Basel). 2021 Apr 28;11(5):138. doi: 10.3390/bios11050138 (PMC8144989; doi:10.3390/bios11050138)
Supplement: Supplementary file 1 [file biosensors-11-00138-s001.zip › biosensors-1170282-sup/SI_Biosensors_MAO_JC.pdf]

Supporting information of: In situ LSPR sensing of secreted insulin in organ-on-chip

María. A. Ortega[1]; Julia Rodríguez-Comas[1]; Ozlem Yavas[2]; Ferrán Velasco-Mallorquí[1]; Jordina Balaguer-Trias[1], Victor Parra[1]; Anna Novials[3,4]; Joan M. Servitja JM[3,4]; Romain Quidant[2,5,6]; Javier Ramón-Azcón[1,6]

1 Biosensors for Bioengineering Group, Institute for Bioengineering of Catalonia (IBEC), The Barcelona Institute of Science and Technology (BIST), Baldiri I Reixac, 10-12, 08028 Barcelona, Spain

2 Plasmon Nano-Optics Group, ICFO-Institute for Photonics Sciences, The Barcelona Institute of Science and Technology, Castelldefels, 08860 Barcelona, Spain

3 Diabetes and Obesity Research Laboratory, Institut d'Investigacions Biomèdiques August Pi i Sunyer (IDIBAPS), 08036 Barcelona, Spain.

4 Centro de Investigación Biomédica en Red de Diabetes y Enfermedades Metabólicas (CIBERDEM), 28029 Madrid, Spain

5 Nanophotonic Systems Laboratory, Department of Mechanical and Process Engineering, ETH Zurich, Zurich, 8092 Switzerland

6 ICREA-Institució Catalana de Recerca i Estudis Avançats, 08010 Barcelona, Spain

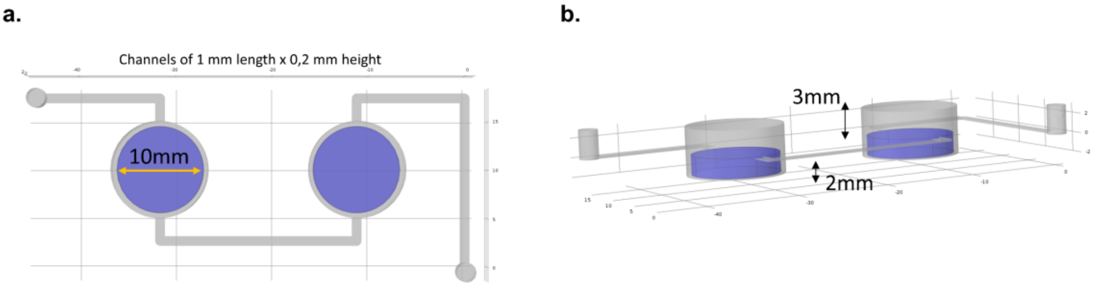

Figure S1. Schematic representation of dimensions of IOC device. (a) Dimensions of channels and chambers where CMC-islets is located. (b) 3D overview of the two-layers of PDMS microfluidic IOC device.

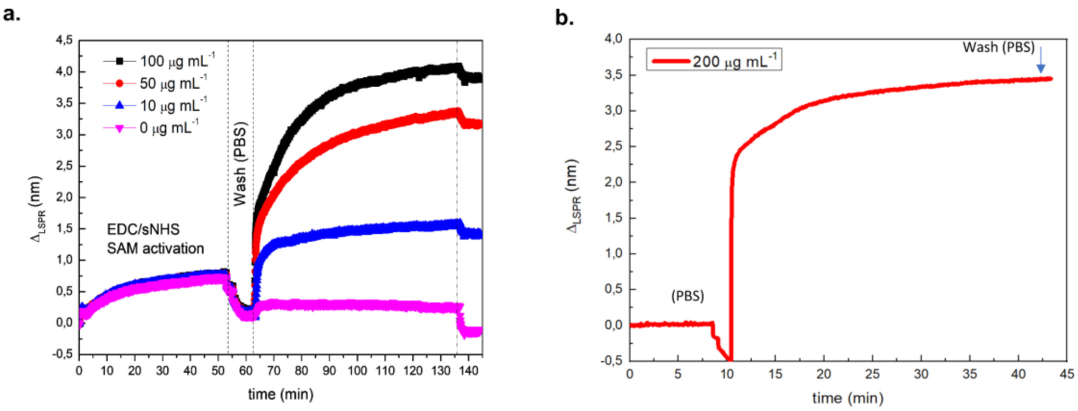

Figure S2. Raw data of the sensograms obtained during optimization of capture monoclonal anti-insulin antibody attached on LSPR gold antennas for (a) 0, 10, 50 and 100 μg mL^{-1} antibody concentrations and (b) 200 μg mL^{-1} antibody concentration respectively. 200 μg mL^{-1} concentration raw data curve showed a faster binding kinetics reaching saturation after ~ 40 min after injection.

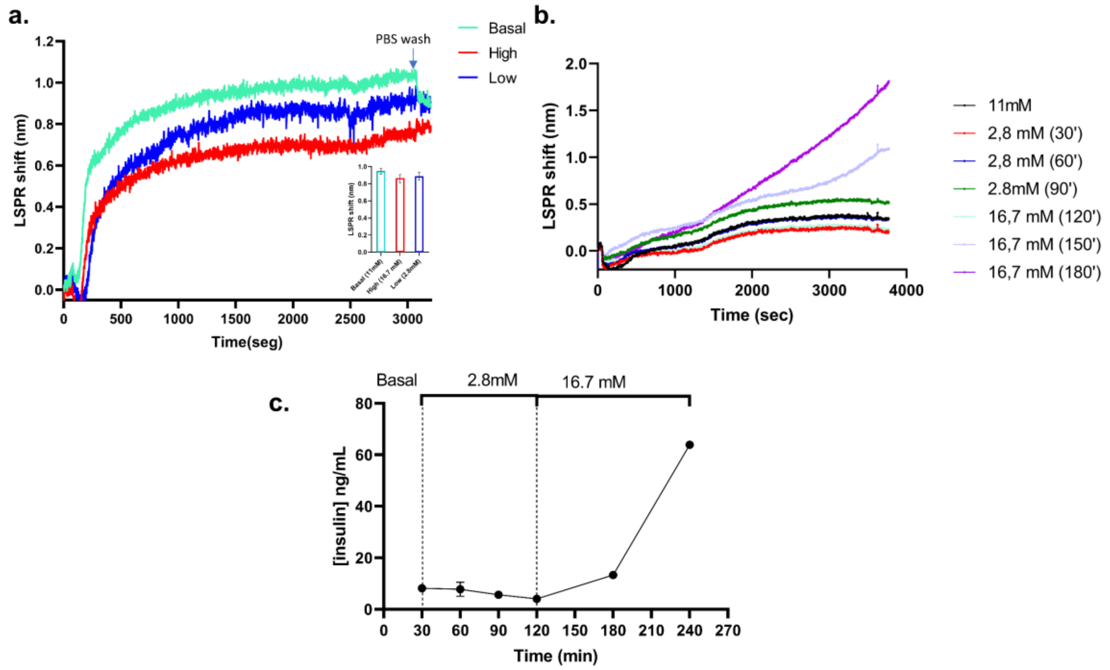

Figure S3. (a) Sensograms showing the matrix effect observed for the relevant glucose concentrations used in IOC experiments. (b) Sensograms (Raw data) obtained for insulin detection every 30 min of secretion using low (2.8 mM) and high (16.7 mM) glucose stimulation regimes. Signals were acquired from different channels of the microfluidics LSPR chip. (c) Insulin detection profile from a second IOC device analyzed by ELISA technique every 30 min stimulated with KRBH buffer at low and high glucose concentration, respectively.
